# Online Health (Mis)Information: The Role of Medical Students

**DOI:** 10.3390/healthcare11010021

**Published:** 2022-12-21

**Authors:** Dalia Y. M. El Kheir, Zainab T. Al Awani, Zainb A. Alrumaih, Majd A. Assad

**Affiliations:** 1 Department of Family and Community Medicine, College of Medicine, Imam Abdulrahman Bin Faisal University, Dammam 31146, Saudi Arabia; 2 College of Medicine, Imam Abdulrahman Bin Faisal University, Dammam 31146, Saudi Arabia

**Keywords:** medical students, social media, online information reliability, physician self-promotion, medical ethics

## Abstract

The public perceive social media as a convenient source of health information. Some physicians might use this to enhance their visibility and market value. In this study, we aimed to assess medical students’ awareness of regulations for dispersion of health-related information on social media and physicians’ online self-promotional activities. A cross-sectional study was conducted among undergraduate medical students from the 3 largest administrative regions of Saudi Arabia: Central, Western, and Eastern regions. Data was collected between February–July 2020 via online distribution of a self-administered questionnaire. Results showed that: (a) a total of 730 medical students participated; (b) about half of respondents were unsure or unaware of guidelines of both, online posting of medical information and physicians’ online self-promotional activities (343/47% and 385/52.7%, respectively); (c) 610 (83.6%) students supported that healthcare providers report accounts sharing unreliable health information. Physicians’ online promotional activities, and posting about successful cases, might shift physicians’ focus from patient care to becoming more popular online. Care should be taken not to breach essential professional and ethical principles, such as protecting the confidentiality and privacy of patients. Raising awareness among patients and physicians, current and future ones, of the regulations governing these online health related interactions is imperative.

## 1. Introduction

In recent years, the utilization of social media (SM) among physicians, medical students and health care institutions has expanded significantly [1]. Physicians use SM to obtain updated medical information, disseminate their clinical knowledge to the public, and conduct medical researches. The expanding role of SM in the healthcare arena has raised some ethical concerns, such as protecting patients’ privacy and confidentiality, which require further examination and implementation of adequately suited guidelines [2].

SM has a very broad and continuous evolving definition. It includes social networking sites, professional networks, media sharing platforms, content production, knowledge and information aggregation, and virtual and augmented reality [3]. According to the last update in 2021, the number of users of SM platforms has increased to reach up to 4.48 billion people worldwide [4]. Currently, increasing numbers of the public perceive SM as a convenient source for obtaining health related information [3,5]. However, the quality of such online information is questioned [6,7,8]. Furthermore, SM was observed to facilitate the spread of misinformation, such as recent conspiracy theories about COVID-19 and related anti-vaccination campaigns [8,9,10]. This adversely influences people’s health decisions [8,9,10,11,12]. Healthcare practitioners have a vital role in improving this new information source, by distributing reliable information and guiding patients to use trustworthy resources [13].

Many doctors nowadays use their SM accounts to disseminate evidence-based health care information [3]. However, when such a communication venue is established, some physicians use it as an opportunity to enhance their visibility and market some services and products [3]. Medicine as a profession is unique in its responsibilities, tradition, and privileges [14], and developing and sustaining a mutual trust between physicians and patients is a cornerstone of the profession. Some have justified that physicians need to promote for themselves, especially with the new global move toward healthcare privatization, while others fear this might come at the expense of patients’ care and their trust [15,16].

Saudi Arabia is currently witnessing a rapid increase in modern technology use in healthcare service delivery, such as the use of different SM platforms and mobile applications to communicate with patients about laboratory testing results, imaging reports, and conducting virtual clinics [17]. An emerging challenge is also physicians’ perceived need for self-promotion through SM especially in the presence of the current local move toward privatization in the Saudi healthcare sector [18,19]. Internationally, the American Medical Association (AMA) and the American Telemedicine Association (ATA) have published practice codes and guidelines, for online interaction with the public, which physicians and regulating bodies may find useful [20,21]. It is expected that the use of SM in healthcare will continue and even increase in the future. Raising awareness among patients and physicians, current and future ones, of the regulations governing these online health related interactions is imperative. Thus, it is necessary to assess medical students’ knowledge regarding SM use for healthcare purposes, online self-promotion activities, and their related regulating guidelines. 

Although there are some recent studies on the topic that describe students’ general pattern of SM use and its frequency [22,23,24], there is lack of studies, especially in the middle east, that addresses medical students’ SM use in health-related purposes, or whether these future doctors are aware of the guidelines regulating online information sharing and physicians’ self-promotion activities. Hence, this study aimed to assess medical students’ awareness of rules and regulations concerning dispersion of health-related information on SM, and their perception regarding physicians’ online self-promotion activities and related governing guidelines.

## 2. Methods

A cross sectional study was conducted among undergraduate medical students from the 3 largest administrative regions of the Kingdom of Saudi Arabia (KSA), namely: Central, Western, and Eastern regions. Data was collected between February-July 2020 via an online, structured, pre-tested, self-administered questionnaire, which was developed through Google forms.

**Figure 1:**
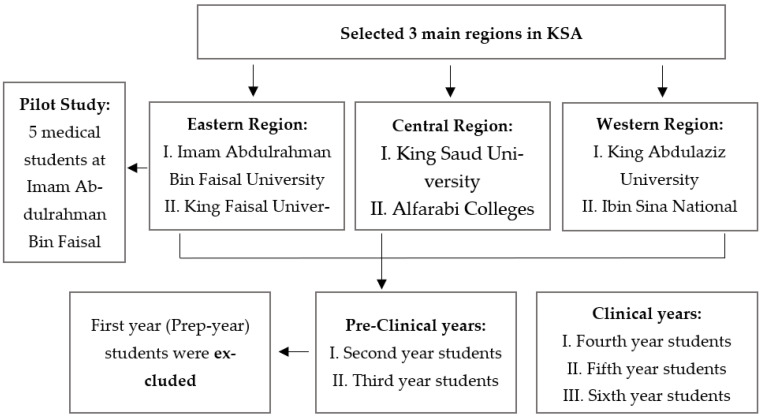
Data Collection Process.

The target population for this study were undergraduate, pre-clinical and clinical, male and female, 2nd–6th years medical students enrolled in any of the following six major medical training institutions: Imam Abdulrahman Bin Faisal University (IAU), King Faisal University, King Saud University, Alfarabi Colleges, King Abdulaziz University, and Ibin Sina National College. These institutions were chosen as they are the largest medical training facilities in the respective targeted KSA regions. Preparatory, 1st year undergraduate medical students in KSA have typically not yet started their medical training, and as such were excluded from this study. Student data collectors were enrolled from all of the above six institutions and trained to distribute the study survey through key student SM platforms in their institutions. The data collection process is summarized in Figure 1.

The study sample size was calculated as a minimum of 384 medical students, using Epi Info TM Software, with 95% confidence interval, *p*-value of 0.05 and a 5% margin of error. The final collected sample was increased to 700 undergraduate medical students to ensure adequate sampling of students in each of the 3 targeted regions of KSA.

The study questionnaire included questions about key demographic data such as age, sex, nationality, academic year, region of studying, and about undergraduate students’ use of SM, purpose of that use, hours spent on SM, number of SM accounts, and SM platform most used. The previously mentioned variables were assessed using multiple choice format questions except for SM use which was assessed using a yes/no format question. In addition, a 5-point Likert scale (strongly agree, agree, neutral, disagree, and strongly disagree) was used to assess students’ perspective on the reliability of online health related information, and physicians’ self-promotion activities on SM. Regarding the reliability of online health information section, it included eight questions which were divided into knowledge, attitude, and future practice. Two questions were assessing the knowledge, four questions for attitude, and two questions for the future practice. On the other hand, physicians’ self-promotion activities section included six questions, one question was assessing the knowledge, three questions for attitude, and two questions for the future practice.

The research team constructed the study questionnaire after review of the relevant literature on the topics investigated [1,25,26]. An independent expert then reviewed it for comprehensiveness and validity of content. To evaluate the questionnaire’s acceptability and length, it was piloted, in January 2020, on an independent sample of 5 undergraduate medical students. Feedback from the pilot students was used to improve the survey questions. Cronbach’s Alpha was used to determine the internal consistency and reliability of the survey. Cronbach’s Alpha estimate was 0.84 showing high internal consistency for the 14 main items tested in our questionnaire (without the demographic variables). 

Common method bias (CMB) and variance, at the design and implementation stages, was reduced through the following steps:

1. Ensuring clarity and accuracy of the wording of survey questions, in addition to pre-testing the questionnaire (piloting) for further item refinement.
2. Different item formats, such as positive, action, neutral, or questioning statements were used in order to limit extreme and biased (non-)conformity response styles.
3. In addition to 2 above, different scale response options were used (yes/no, and a 5-point Likert scale options). This aims to reduce similarity in our multi-item scale measurements and prevents using of one item’s response to reveal the answer to another related question.
4. To encourage them to be honest and open about their responses, participants were assured of anonymity and strict data confidentiality before commencing data collection.

In addition, the Harman’s single factor test for CMB, using exploratory factor analysis, was performed. The total variance explained by a single factor is 27.523% which is less than 50% suggesting that CMB does not affect our data, hence our results.

For the purpose of the current analyses, the 5 categories of the Likert scale were condensed to 3 categories as follows: strongly agree was combined with agree in a new category called agree, similarly, strongly disagree was added with disagree forming a new category called disagree, the neutral category was unchanged. The collected data were coded and analyzed using SPSS [the statistical package for social sciences IBM SPSS Statistics for Windows, version 28 (IBM Corp., Armonk, NY, USA)], and results as frequencies, percentages and summary statistics, are presented in tables. The final analyses presented in this article exclude pilot data. Chi-square test of significance, with cut-off value of significance *p* ≤ 0.05, was used to test the association of medical students’ academic level, 2nd to 6th years, with their awareness of guidelines for both online sharing of health-related information and self-promotion activities, in addition to the relationship of the latter with medical students’ possible future intention of use of SM influencers to advertise for them.

This study was ethically approved by the Institutional Review Board (vide letter no. IRB-UGS-2019-01-211). All study participants provided informed consent prior to data collection. Respondents were assured that the study data would only be accessed by the research team, used exclusively for research purposes, and that they were free to withdraw from the study at any time.

## 3. Results

A total of 730 students participated and completed our survey. Almost all respondents were Saudis 710 (97.3%) and the majority, 422 (57.8%), were aged between 21–23 years. Females represented half of the sample 371 (51%), and two-thirds of respondents were from Eastern region 463 (63.4%). Additionally, the study included medical students studying at the pre-clinical, 269 (36.8%), and clinical years, 461 (63.1%). Participants’ demographic data are displayed in Table 1.

Table 2 details the pattern and frequency of our respondent’s SM use. Almost all students, 716 (98.1%), use SM with 458 (62.7%) of them having more than one account in multiple sites. About three quarters of the respondents spend between 1–3 h and 4–6 h on SM daily; 292 (40%) and 305 (41%), respectively. WhatsApp ranked number one most used SM platform, indicated by 594 (81.4%) students, followed by Twitter (511, 70%), Snapchat (463, 63.4%), and Instagram (409, 56%).

Our sampled undergraduate students were asked their opinion (agree/disagree/neutral) regarding several aspects of online health related information sharing and relevant guidelines (Table 3). There was a high level of agreement, ranging between 79.7% and 87.4%, among our respondents on almost all statements queried. The 2 exceptions with markedly lower level of agreement between respondents, range of 53–55.9% agreement, respectively, were the statements: (a) “I am aware of guidelines addressing the dispersion of reliable medical in-formation online”, and (b) “I am aware of the reporting system for accounts found sharing unreliable information” (Table 3). The relationship between medical students’ study level, 2nd–6th years, and their knowledge of guidelines addressing sharing of reliable medical information online was statistically significant (*p* value = 0.023; Table 3). 

Participating medical students also answered several questions discussing physicians’ self-promotion activities on SM (Table 4). Overall, between 30–40% of students were neutral, or unsure, of all aspects introduced about the topic of physicians’ online self-promotion. Starting with awareness of the rules and regulations about online self-promotion, 345 (47.2%) students agreed they were aware of such guidelines, while 206 (28.2%) were neutral and 179 (24.5%) unsure. A similar pattern was seen in the remaining 5 statements about the topic (Table 4) where the numbers of those agreeing and those unsure were very close.

The relationship between medical students’ study level, 2nd to 6th years, and their awareness of the rules and regulations of online self-promotion is not statistically significant (*p* value = 0.59). On the other hand, the relationship between students’ possible future intention of paying SM influencers to advertise for them and their awareness of online self-promotion regulations is statistically significant (*p*-value = 0.000; Table 4). 

## 4. Discussion

This study aimed to explore medical students’ awareness, perception and future practice regarding different aspects of health-related information on SM. Nowadays, patients are increasingly reliant on SM as an easily accessible source of medical and health related information [27]. However, the published scientific literature has shown SM to be one of the less trusted sources of health information [28]. It is an important responsibility of physicians to make this new information source a more reliable evidence-based platform, by engaging with, contributing to, and correcting the medical information shared in it [29,30].

In KSA instant messaging applications are commonly used in telemedicine [31]. Nevertheless, globally these modalities do not have clear guidelines governing their use in healthcare [32]. The American Medical Association (AMA) has described some ethical codes for using SM in healthcare, including: the need for providing the source of information when posting online, sharing credible and relevant health information resources, discouraging the behavior of sharing medical information without ensuring its reliability and validity, and supporting the provision of adequate and reliable information to online consultations through SM [26]. We found that overall, our students had good knowledge of these ethical codes, with 87.4%, 79.7%, 85.4%, and 83% of participants agreeing with these codes, respectively. 

However, when it came to guidelines regulating the process of online posting of medical information, about half of our respondents were either unsure or unaware of such guidelines. A similar result was seen regarding students’ awareness about reporting systems for online accounts sharing unreliable or incorrect health information. Nonetheless, 83.6% of participants think healthcare providers should report accounts sharing unreliable health information, and another 86% would even address their fellow colleagues who might have posted erroneous health related information. This confirms the results of a recent study where 70% of the surveyed Saudi physicians felt an obligation to correct inaccurate online information [31]. The AMA puts the responsibility of reporting physicians’ unprofessional online content, to the appropriate authorities, on their fellow colleagues [26]. Nevertheless, if physicians are not sure or not aware of such authority, then they might fail to properly report such incidents, in which case corrective action might be delayed or not take place.

Of note here is the fact that 53% of our surveyed medical students declared they are aware of the guidelines governing online sharing of medical information. It might be that these students are referring to their knowledge of the basic principles of ethics taught in their undergraduate medical curriculum [33,34] or their own judgment of what is right and what is wrong. Medical ethics principles regulating traditional medical care provision might still apply to SM use in healthcare. Nevertheless, these new online healthcare modalities have special ethical and legal requirements which call for targeted specific training of current and future physicians alike [35]. In fact, the Saudi National Health Information Centre recently announced the telemedicine regulations in the country, in 2018 [36]. Physicians are urged to respond to patients’ SM enquiries using standard general responses and employ encrypted regulated channels to provide online healthcare [37,38]. Practice codes and guidelines developed by some international organizations, such as the AMA and the ATA are useful resources for physicians and regulating bodies [20,21].

When asked about the regulations governing physician’s online self-promotion, more than half of our surveyed medical students were either unsure or unaware of any such guidelines. An additional 40% of respondents were unsure whether online self-promotion by posting about one’s own successful cases was unethical or whether they would do the same as future physicians. Nowadays, patients obtain health information and interact with physicians using SM platforms [37,39,40,41]. Hence, SM sites have become a big marketing arena and can increase the public’s recognition of physicians and healthcare services, one prominent example is online promotion of cosmetic surgery seen in KSA and internationally [37,39,40,41]. It has been shown that such strategy of online advertisement has recently increased, benefitting from the public’s trust in the product and service information provided by friendly SM influencers [41]. It is expected that the use of SM in healthcare will continue and even increase in the future. Raising awareness among patients and physicians, current and future ones, of the regulations governing these online health related interactions is therefore imperative.

Furthermore, only a third of our participating medical students believed that physicians’ success depends on their ability to promote themselves on SM, and 20.5% would pay a SM influencer to advertise their work as future physicians. The findings of SM marketing research revealed that attitudes of consumers are not readily affected by their engagement with influencers on SM [41]. Moreover, research had also indicated that due to their online promotional activities, physicians’ focus on patient care might shift to increasing their online popularity [38,39,40,41,42].

From another perspective, 39.2% of our sampled medical students admitted they would, as future physicians, post about their own successfully treated cases online. This might prove beneficial for some healthcare privatization schemes. However, the benefits of such actions by physicians must be carefully weighed against the potential risks. Such behavior might breach essential professional and ethical principles, such as protecting the confidentiality and privacy of patients [43]. The ease of going online and publishing content has its implications on good physicians’ practice, and different guidelines on the professional use of SM have been released [44,45]. For instance, a healthcare worker could unintentionally post a picture of a patient, or a patient’s chart while taking a picture of their lunch, unaware of the chart in the background [46]. This calls upon medical students and healthcare workers to exercise extreme caution when posting anything online, as this might expose them to disciplinary action. Disciplinary actions may range from receiving a letter of reprimand, mandated education, receiving a monetary fine or community service, to restriction of license, suspension of license or probation [47].

Furthermore, online self-promotion was regarded by 56.3% of our medical students as having potential to raise awareness about the medical field, and thereby improving the public’s knowledge and helping consumers better navigate an increasingly complex healthcare landscape. Notwithstanding, care should be taken when engaging in such promotional activities as previous research has shown that some physicians have used SM to attract patients and gain profit by posting more self-promotional than educational content [22,37,48].

A major strength of the present investigation is that it included undergraduate students from the main medical training institutes of 3 major KSA regions. This would enhance the generalizability of our findings. However, the descriptive nature of our study might be considered a limitation as it restricts a more nuanced understanding of students’ perceptions, attitudes, and interpretation of the study statements and proposed practice situations. Notwithstanding, we believe that our results highlight a crucial and still relatively deficient area in medical students’ training and preparation for future practice. Our study examines in some depth medical students’ awareness and views of current day physicians’ online presence and interaction with the public. Students’ knowledge and opinions are then projected and linked with their anticipated future clinical practice activities. We believe the current analysis to be one of the first in KSA, and internationally, to report similar results among undergraduate medical students.

It is imperative for medical students to receive targeted education regarding proper use of SM in healthcare, dissemination of health information via SM platforms, and concerned policies and guidelines. Therefore, medical schools should take into consideration incorporating digital health literacy training programs into the medical curriculum [49,50]. Such a curriculum may also address emerging issues such as physicians’ online self-promotion and related governing policies and guidelines.

## 5. Conclusions

Following the COVID-19 era and the increased rates of telemedicine and SM use in healthcare delivery, it is expected that these online healthcare modalities will become permanent features of current and future healthcare systems. Undergraduate medical students, our future medical workforce, are inadequately prepared to handle the challenges presented by these new modalities. The reliability of online health information and physician’s self-promoting activities are two emerging challenges that our students will face during their future clinical careers. Therefore, timely identification of medical trainees’ related knowledge, skill level, and training needs regarding these two aspects is essential, and would facilitate enhancement of undergraduate medical education. Future research to identify suitable training programs, with robust didactic features, is warranted. 

## Figures and Tables

**Table 1:** Demographic data of study participants (n = 730).

Variables	n	%
**Age in years**		
18–20	164	22.5
21–23	422	57.8
42–26	138	18.9
26 or more	6	0.8
**Gender**		
Female	372	51
Male	358	49
**Nationality**		
Saudi	710	97.3
Non-Saudi	20	2.7
**Current region**		
Eastern	463	63.4
Central	143	19.6
Western	124	17
**Academic Year**		
2nd year	131	17.9
3rd year	138	18.9
4th year	186	25.5
5th year	144	19.7
6th year	131	17.9

**Table 2:** Social media use by study participants (n = 730).

Variables	n	%
**Do you use social media websites?**		
Yes	716	98.1
No	14	1.9
**Do you have one account or more on social media?**		
Yes, more than one account in more than one site	458	62.7
Yes, more than one account in only one site	97	13.3
Yes, one account in only one site	173	23.7
No, I do not have an account	2	0.3
**How many hours do you spend on social media daily?**		
Less than 1 h	26	3.6
1–3 h	292	40
4–6 h	305	41.8
More than 6 h	173	23.7
**Which social media platform do you use the most? (multiple answers allowed)**		
Whatsapp	594	81.4
Twitter	511	70
Snapchat	463	63.4
Instagram	409	56
Telegram	154	21.1
Youtube	32	4.4
Facebook	28	3.8
Tiktok	5	0.7
Others	6	0.8
**For what purpose do you use social media? (multiple answers allowed)**		
Personal	675	92.5
Educational	552	75.6
Health purposes	275	37.7
Business	61	8.4
Entertainment	60	8.2
Others	5	0.7

**Table 3:** Medical students’ view on the reliability of health information shared on social media (n = 730).

Statement	Disagree	Neutral	Agree
I am aware of any guidelines addressing the dispersion of reliable medical information online *	94 (12.9%)	249 (34.1%)	387 (53%)
I am aware of the reporting system if there were any accounts found sharing unreliable information	143 (19.6%)	179 (24.0%)	408 (55.9%)
I think it is important for health care professionals to provide the source of any information they post online	9 (1.2%)	83 (11.4%)	638 (87.4)
As a health care provider, I think it is essential to report any account that provides unreliable health-related information	10 (1.4%)	110 (15.1%)	610 (83.6%)
I think it is essential to give patients reliable resources in order for them to gain knowledge and search about their medical conditions	22 (3.0%)	126 (17.3%)	582 (79.7%)
I think it is unethical to post unreliable medical information in my social media accounts	23 (3.2%)	83 (11.4%)	624 (85.4%)
If I found one of my colleagues posting incorrect information about a certain medical condition in their social media accounts, I would feel responsible to tell them the true information and ask them to delete/correct the post	12 (1.7%)	89 (12.2%)	629 (86.1%)
As a future physician, I would make sure to provide adequate and reliable information through social media for my patients if they ask about their illness	13 (1.8%)	110 (15.1%)	607 (82.95%)

* The relationship between medical students’ study level, 2nd–6th years, and their knowledge of guidelines addressing sharing of reliable medical information online was statistically significant (*p* value = 0.023).

**Table 4:** Medical students’ view on physicians’ online self-promotion activities (n = 730).

Statement	Disagree	Neutral	Agree
I am aware of the rules and regulations about online self-promotion *	179 (24.5%)	206 (28.2%)	345 (47.2%)
I find it unethical to promote for myself by posting about successfully treated cases on social media	158 (21.6%)	296 (40.5%)	276 (37.8%)
I believe that successful doctors are the ones who know how to promote for themselves on social media	290 (39.7%)	209 (28.6%)	231 (31.7%)
I find that online self-promotion helps in spreading awareness about the medical field	85 (11.6%)	234 (32.1%)	411 (56.3%)
As a future physician, I would post about successful cases I treated online	162 (22.2%)	282 (38.6%)	286 (39.2%)
As a future physician, I would pay for social media influencer to advertise for me ^§^	408 (55.9%)	173 (23.7%)	149 (20.5%)

* The relationship between medical students’ study level, 2nd to 6th years, and their awareness of the rules and regulations of online self-promotion is not statistically significant (*p* value = 0.59). § The relationship between students’ possible future intention of paying social media influencers to advertise for them and their awareness of online self-promotion regulations is statistically significant (*p*-value = 0.000).

## Data Availability

The datasets used for the current study are available from the corresponding author upon reasonable request.

## Abbreviations

SM	Social Media
KSA	Kingdom of Saudi Arabia
AMA	American Medical Association
ATA	American Telemedicine Association
IAU	Imam Abdulrahman Bin Faisal University
CMB	Common Method Bias
SPSS	Statistical Package for Social Sciences
IRB	Institutional Review Board
